# Setting a stage: Inflammation during preeclampsia and postpartum

**DOI:** 10.3389/fphys.2023.1130116

**Published:** 2023-02-23

**Authors:** Owen Herrock, Evangeline Deer, Babbette LaMarca

**Affiliations:** ^1^ Department of Pharmacology and Toxicology, University of Mississippi Medical Center, Jackson, MS, United States; ^2^ Department of Obstetrics and Gynecology, University of Mississippi Medical Center, Jackson, MS, United States

**Keywords:** preecclampsia, immune cells, chronic inflammation, pregnancy, postpartum

## Abstract

Preeclampsia (PE) is a leading cause of maternal and fetal mortality worldwide. The immune system plays a critical role in normal pregnancy progression; however, inappropriate inflammatory responses have been consistently linked with PE pathophysiology. This inflammatory phenotype consists of activation of the innate immune system, adaptive immune system, and increased inflammatory mediators in circulation. Moreover, recent studies have shown that the inflammatory profile seen in PE persists into the postpartum period. This manuscript aims to highlight recent advances in research relating to inflammation in PE as well as the inflammation that persists postpartum in women after a PE pregnancy. With the advent of the COVID-19 pandemic, there has been an increase in obstetric disorders associated with COVID-19 infection during pregnancy. This manuscript also aims to shed light on the relationship between COVID-19 infection during pregnancy and the increased incidence of PE in these women.

## Introduction

Preeclampsia (PE) is a multisystem obstetric disorder that presents as new onset of hypertension in conjunction with evidence of end-organ dysfunction beyond the 20th week of gestation (ACOG, 2022). PE is a leading cause of maternal-perinatal morbidity and mortality each year and occurs in about 7% of pregnancies (English et al., 2015). In conjunction with hypertension, PE patients may present with proteinuria, HELLP (Hemolysis, Elevated Liver enzymes, and Low Platelets), or visual disturbances (Roos et al., 2012; ACOG, 2022). The only cure for PE is delivery of the fetal-placental unit, making PE a leading cause of premature birth (Staff et al., 2022). Moreover, the offspring of PE pregnancies are at higher risks for stillbirth, FGR (Fetal Growth Restriction), and additional neonatal complications (Fox et al., 2019). Therefore, the management of PE pregnancies is a delicate balance between maternal and fetal health, which could be attributed to the lack of any major developments or changes in treatments for PE in the last 50 years (Bell, 2010).

PE is a state of chronic inflammation with activation of antigen presenting cells (APC’s), T helper (Th) cells, B cells, and Natural killer cells which contribute to the presentation of PE symptoms during pregnancy (LaMarca et al., 2016; Aneman et al., 2020). Th cells secrete cytokines that activate innate immune cells, induce production of anti-angiogenic factors, and increase sodium conductance in the nephron (LaMarca et al., 2016). B cells produce agonistic antibodies against the angiotensin II type 1 receptor (AT1-AA) which activate the Renin-Angiotensin system and activate killer cells to target AT1-AA marked cells (Campbell et al., 2018). Moreover, innate immune cells contribute to the inflammatory cytokine milieu while contributing to tissue damage in the kidney and placenta (Aneman et al., 2020). Over the COVID-19 pandemic, there has been an increase in the incidence of PE in women that contracted COVID-19 during their pregnancy (Jamieson and Rasmussen, 2022). COVID-19 infection during pregnancy leads to an immune reaction in the decidua; however, this immune reaction appears to change depending on the trimester in which the COVID-19 infection occurs (Juttukonda et al., 2022). Furthermore, studies in women that contract COVID-19 during pregnancy have shown that there are increased markers of placental dysfunction or placental damage (Jaiswal et al., 2021; Schwartz et al., 2022). Inflammation and placental damage following COVID-19 infection could be contributing factors toward the increased incidence of PE in women with a history of COVID-19 infection during their pregnancies (Villar et al., 2021).

**FIGURE 1:**
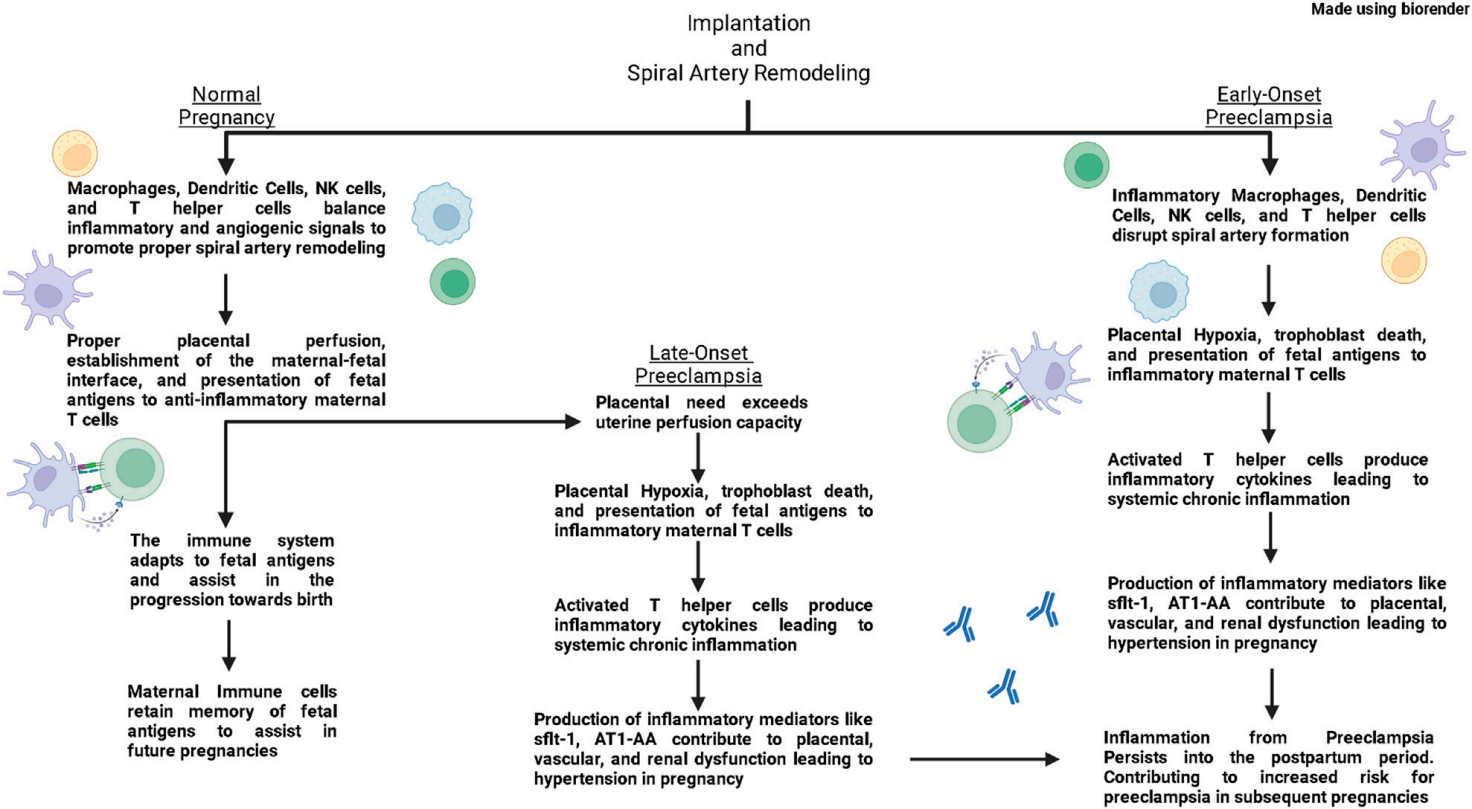
In normal pregnancies, macrophages, dendritic cells, Natural Killer (NK) cells, and T helper (Th) cells produce both inflammatory and anti-inflammatory signals that support vascular remodeling and promote spiral artery remodeling. As pregnancy progresses, antigen presenting cells present fetal antigens to Th cells and polarize them towards a regulatory Th cell phenotype (Tregs). These Tregs then quell immune responses in the placenta and maintain an anti-inflammatory environment through the second trimester. As a normal pregnancy reaches its end, maternal immune cells produce inflammatory signals that help signal the uterus for delivery. In the postpartum period, maternal immune cells retain memory of fetal antigens to help in future pregnancies. In early-onset preeclampsia, inflammatory macrophages, dendritic cells, NK cells, and Th cells disrupt spiral artery formation leading to placental hypoxia. Hypoxia leads to cell death which leads to placental dysfunction and release of fetal antigens. Antigen Presenting Cells present these fetal antigens and polarize Th cells toward an inflammatory phenotype. Inflammatory Th cells then induce production of inflammatory and anti-angiogenic factors from the placenta and other immune cells which lead to placental, vascular, and renal dysfunction contributing to hypertension in pregnancy. These inflammatory cells from preeclampsia retain memory postpartum, and increase the risk for developing PE in subsequent pregnancies. In late-onset preeclampsia, the spiral artery formation occurs normally, however eventually the needs of the placenta out-weigh the perfusion capacity of the uterus, leading to placental hypoxia and a similar inflammatory environment as seen in early-onset preeclampsia.

While symptoms of PE usually end upon delivery of the placenta, the inflammation persists into the mother’s postpartum period. Research has demonstrated that patients have an increased risk of recurrent PE, hypertension, coronary heart disease and stroke following a PE pregnancy (Wu et al., 2017; Opichka et al., 2021), thereby illustrating the long-term cardiovascular complications caused by PE. Furthermore, the immune system and inflammation has been repeatedly implicated in the pathophysiology of cardiovascular disease during pregnancy and outside of pregnancy (Harrison et al., 2021; Stefanska et al., 2021). Therefore, this review aims to provide insight into the potential role of immune cells and immune mediators in contributing to PE and cardiovascular disease postpartum of PE. This review aims to highlight clinical studies that have elucidated contributions of the immune system in physiological pregnancy and PE; while also presenting basic science studies that have provided mechanistic insight into how immune mediators impact pregnancy (Figure 1).

## Placental ischemia—The initiating event in preeclampsia pathophysiology

During normal pregnancies, fetal trophoblasts invade the maternal myometrium with the aid of uterine immune cells (Staff et al., 2022). Trophoblasts and immune cells secrete proteases and angiogenic factors to progressively remodel uterine spiral arteries into high-capacity, low-resistance vessels (Albrecht and Pepe, 2020). In PE pregnancies, there is a breakdown in placental perfusion which ultimately results in placental ischemia. Placental ischemia induces the production of inflammatory modulators and anti-angiogenic factors (Zhang, 2018) which contribute to vascular dysfunction in the placenta and peripheral vessels. Furthermore, these circulating placental factors cause endothelial dysfunction and oxidative stress which contribute to hypertension and multi-organ dysfunction as seen in PE (Rana et al., 2019).

PE presents as systolic pressure ≥140 mmHg or diastolic pressure ≥90 mmHg with an accompanying organ dysfunction including elevated 24-h urine protein value of ≥300 mg, urine protein to creatine ratio of ≥0.3, thrombocytopenia, impaired liver function, pulmonary edema, or new-onset headache with or without visual disturbances (Karrar and Hong, 2022). PE can be stratified by the timing of symptom presentation. While these categories of PE are unified by placental ischemia, they are believed to stem from different etiologies. PE that develops prior to 34 weeks of gestation is considered early-onset (EO) PE, PE that develops after 34 weeks of gestation is considered late-onset (LO) PE, and while PE that develops after delivery of the fetal-placental unit is considered postpartum PE (PP-PE) (Raymond and Peterson, 2011; Hauspurg and Jeyabalan, 2022). EOPE is characterized by insufficient invasion of trophoblasts into the endometrium resulting in poor spiral artery remodeling leading to placental ischemia and PE phenotype (Tamas et al., 2022). EOPE is considered the most severe form of PE and causes the highest rates of morbidity and mortality in mother and neonate compared of the PE subsets (Madazli et al., 2014). EOPE mothers are the most likely to have placental pathologies and higher PE recurrence rates compared to LOPE mothers (Li et al., 2014; Fillion et al., 2021). Mothers with both placental pathologies (syncytial knots, placental infarcts, etc.) and severe PE symptoms are more likely to have strokes or develop cardiovascular disease postpartum (Veerbeek et al., 2015). LOPE is associated with normal placentation in early pregnancy, with placental dysfunction occurring later in pregnancy. LOPE is projected to occur when the needs of the placenta are above the perfusion capacity of the uterus leading to placental ischemia and the symptoms of PE (Staff et al., 2022). Even though LOPE is less associated with neonatal morbidity (Staff et al., 2016; Wadhwani et al., 2020), LOPE still predisposes mothers and offspring to develop cardiovascular disease later in life. Moreover, children born of LOPE pregnancies are less likely to be growth restricted. However, they still have increased incidence of cardiovascular disease and stroke in later life compared to children born of normal pregnancies (Kajantie et al., 2009; Touwslager et al., 2012), thereby suggesting that cardiovascular disease in PE offspring is caused by more than just FGR. LOPE is often associated with increased maternal BMI, high weight gain during pregnancy, and other metabolic disorders (Robillard et al., 2019; Robillard et al., 2022). Obesity and metabolic disorders are considered states of chronic inflammation (Esposito and Giugliano, 2004; Khanna et al., 2022), and these metabolic and inflammatory signals may contribute to the increase risk of cardiovascular disease in offspring of women with LOPE. Lastly, there are rare cases where patients develop a PE phenotype after delivery, which is known as PP-PE. Patients with PP-PE have similar placental vasculopathies that resemble LOPE by demonstrating a similar role for placenta dysfunction (Ditisheim et al., 2020; Hauspurg and Jeyabalan, 2022). But, more studies are needed to elucidate the mechanisms that induce postpartum PE.

## Dynamic shifts in the immune system during normal pregnancy

### Implantation and the first trimester of pregnancy

In a normal pregnancy, the maternal immune system must balance immune tolerance of the semi-allogenic fetus with defending the mother from disease. However, disruption of this balance has been associated with developing PE. During pregnancy, women undergo time-dependent shifts in activation of the innate and adaptive immune systems, but dysregulation of the immune system can cause adverse outcomes in both the mother and fetus (Cornelius et al., 2019). In the first trimester, macrophages (Mφ) and Natural Killer (NK) cells are two of the most prevalent cells in the uterus (Jena et al., 2019). Uterine subsets of both NK (uNK) cells Mφ (uMφ) are pivotal in uterine remodeling during pregnancy (Gustafsson et al., 2008; Jetten et al., 2014) (Bazhenov et al., 2019). The uNK cell population have high concentrations of intracellular killer granules, but do not release cytolytic granules and are not efficient in cell killing compared to conventional NK cells (Faas and de Vos, 2017). uNK cells have higher expression of Killer cell Immunoglobulin-like Receptors than conventional NK cells; however, uNK cells lack FcγRIII (CD16), which may contribute to their reduced killing capacity in normal pregnancies (Koopman et al., 2003). CD16 recognizes the FC region of IgG antibodies and is responsible for antibody induced cellular cytotoxicity (Yeap et al., 2017). uMφ are unique compared to peripheral macrophages (Houser et al., 2011); however, uMφ resemble alternatively activated M2s phenotype which produces lower levels of the inflammatory IL-1 while producing higher levels of the anti-inflammatory IL-10 compared to inflammatory M1s (Mizuno et al., 1994; Gough et al., 2001). These uMφ are also important in anti-microbial defense in the uterus and clearing cellular debris during pregnancy (Thiruchelvam et al., 2013).

During embryo implantation, the uterine immune cells help to induce vascular remodeling while initiating processes to promote fetal tolerance. uNK cells produce copious amounts of interferon gamma (IFN-γ) which destabilizes vascular endothelial cells and initiates uterine artery remodeling (Ashkar et al., 2000). This uNK cell derived IFN-γ also activates uMφs which allow them to assist in vascular remodeling. These uNK cells and Mφs migrate and localize to fetal trophoblasts (Helige et al., 2014). The uNK cells and Mφs then produce proteases to destabilize vascular smooth muscle (Naruse et al., 2009; Smith et al., 2009) and angiogenic factors (Ferrante et al., 2013; El-Azzamy et al., 2018) that promote growth of new blood vessels and maturation of existing blood vessels. During this critical stage of implantation, uterine stromal cells and uterine dendritic cells (uDCs) work to prevent excessive vascularization during implantation. Uterine stromal cells are also important cytokine producers and cross-talk with uDCs during pregnancy. Uterine stromal cells produce granulocyte-colony stimulating factor (G-CSF) which appears to increase IL-1β from uDCs (Shao et al., 2020). In the short term, this may contribute to uDCs producing soluble Flt1, which binds soluble vascular endothelial growth factor (VEGF) and serves as a control for angiogenesis during pregnancy (Plaks et al., 2008).

IFN-γ from uNK cells also induces uMφ′s to express HLA-II, which is an important in inducing T helper (Th) cell activation (Xie et al., 2005). T helper (Th) cells are CD4+ T cells that are crucial for both directing immune responses and helping to orchestrate transitions in pregnancy. Th1 cells are characterized by the expression of the transcription factor Tbet, and help with uterine remodeling during the first trimester (Faas and De Vos, 2018). Th1s and uNK cells also produce Tumor Necrosis Factor (TNF)-α which prevents excessive invasion of trophoblasts into the uterus through activating the NF-κB pathway (Todt et al., 1996; Torchinsky et al., 2003). Inhibiting IFN-γ in early pregnancy prevents successful uterine artery remodeling therefore causing fetal demise (Ashkar et al., 2000), suggesting roles for Th1s and uNK cells to promote vascular remodeling in pregnancy through IFN-γ production. Overall, uNK cells, uMφs, uDCs, and Th1s cells carefully collaborate to promote the formation of the maternal-fetal interface during the first trimester. Moreover, inflammatory and anti-inflammatory mechanisms are important during implantation in the first trimester.

### Second trimester of pregnancy

The transition from early to mid-pregnancy is accompanied by a shift from a pro-inflammatory to an anti-inflammatory uterine environment. DCs are regulate vascularization during the first trimester however DCs are also professional APC’s that internalize antigens and present them on major histocompatibility complex-II in order to activate Th cells. In pregnancy, DCs help transition from the early pregnancy pro-inflammatory Th1 environment to the middle pregnancy anti-inflammatory Th2 environment. Cross-talk between uterine stromal cells and uDCs eventually promotes uDCs to express co-stimulators of Th cell activation CD80 and CD86 (Shao et al., 2020). Moreover, these uDCs polarize Th cells towards a Th2 phenotype *in vitro*. Negishi et al. (2012) found that DCs that express Dendritic Cell Inhibitory Receptor 2 (33D1+) DCs slowly increase in number during the first trimester and these 33D1+ DCs promote the switch from Th1s to Th2s in middle pregnancy. Further supporting a role for uDCs to induce the pivot from the inflammatory environment needed for implantation towards the anti-inflammatory Th2 environment needed for pregnancy tolerance. Mφs are another important APC that help with clearing cellular debris and help polarize Th cells towards the appropriate Th subtype to promote fetal tolerance in pregnancy. Specifically, T cell immunoglobulin mucin-3 (Tim-3^+^) uMφ help polarize Th cells towards anti-inflammatory Th2 and Treg phenotypes (Li et al., 2022). Tim-3^+^ blockade lead to decreased fetal viability and fetal growth restriction, further implicating Mφ in promoting fetal health during pregnancy. Together these studies show that uDCs and uMφ help to promote an anti-inflammatory environment in the transition towards the second trimester of pregnancy.

Th2 cells begin to increase near the end of the first trimester and continue to predominate over Th1 cells through the rest of the pregnancy (Saito et al., 1999). Th2 cells are producers of the cytokine IL-4, which prevents Th1 cells proliferation and promotes a Th2 profile in normal pregnancies (Lazarski et al., 2013). IL-4 also inhibits IFN-γ production, which reduces NK cell activation and promotes fetal tolerance (Zissler et al., 2016). Th2 cells also promote B cells to produce protective “asymmetric” antibodies during pregnancy (Zenclussen et al., 2001). These asymmetric antibodies bind fetal antigens on the placenta, but do not activate killer cells. Asymmetric antibodies effectively block the fetal antigens from being found by the maternal immune system. Therefore asymmetric antibodies protect paternal antigens and the fetus from killer immune cells while promoting immune tolerance in pregnancy (Gutierrez et al., 2005).

In normal pregnancy, paternal antigen-specific Treg cells expand in circulation and in the uterus to promote fetal tolerance (Kahn and Baltimore, 2010; Shima et al., 2015; Huang et al., 2020). Tregs are known as important producers of IL-10 during pregnancy (Cheng and Sharma, 2015). IL-10 reduces antigen presentation co-stimulatory molecules by APCs which downregulates the ability of APCs to produce IL-12, which is an important stimulator of NK cells and Th1s (Moore et al., 2001; Zundler and Neurath, 2015). Treg cells also constitutively express CTLA4 (Cytotoxic T-Lymphocyte Associated-protein), which reduces the ability of APCs to activate new Th cells and prevent new inflammatory responses (Qureshi et al., 2011). Therefore, Tregs use IL-10 and CTLA4 to prevent new Th1 polarization. IL-10 also protects nitric oxide production pathways (Gunnett et al., 2002) and prevents vascular dysfunction from endogenous vasoconstrictors (Didion et al., 2009).

### Third trimester of pregnancy and parturition

As pregnancy reaches its end, parturition is characterized by a drastic shift towards an inflammatory phenotype (Leimert et al., 2021). Two to four weeks prior to parturition are characterized by a shift from pregnancy maintenance to preparation for labor (Stelzer et al., 2021). During this period, IL-6 and IL-1β promote the production of prostaglandins, endothelin-1, and cyclooxygenases which induce uterine contractions during labor (Ashkar et al., 2000). This inflammatory phenotype may in part be due to the progressive decrease in circulating progesterone and Progesterone Induced Blocking Factor (PIBF) that occurs as normal pregnancies progress to parturition (Polgar et al., 2004; Hudic et al., 2016). Progesterone and PIBF both inhibit lymphocyte proliferation and inflammatory cytokine production, which potentially implicates them as important anti-inflammatory agents in the earlier portion of the pregnancy (Fedotcheva et al., 2022). Near the 37th week of pregnancy, PIBF decreases triggering increases in IL-1β and IL-6 along with decreases in the anti-inflammatory cytokines IL-1Ra and IL-9 (Polgar et al., 2004; Jarmund et al., 2021). Uterine levels of IL-6 appear to be important to induce the onset of labor, as IL-6 deficient mice experience delayed labor compared to controls (Gomez-Lopez et al., 2016). As IL-6 is produced by many cell types, multiple cellular sources of IL-6 likely contribute to the IL-6 needed for parturition. Among major IL-6 producers are Mφs which are sensitive toward changes in estrogen and progesterone during pregnancy (Mendoza-Cabrera et al., 2020). At term, uMφs invade the decidua and cervix and produce reactive oxygen species and TNF-α which lead promote cervical ripening (Hamilton et al., 2012). At this time, uterine levels of the inflammatory Th9 and Th17 subsets increase (Gomez-Lopez et al., 2016). However there has not been a direct link drawn between uMφs and the increase in inflammatory Th subsets. Moreover, the precise mechanisms that induce the shift towards parturition have not been fully elucidated and more studies are needed to tease at the role of the immune system during parturition.

## The chronic inflammatory environment in preeclampsia

The immune system during PE is in a state of chronic inflammation (Cornelius et al., 2019; Aneman et al., 2020; Stefanska et al., 2021). Patients with PE have activation of both the innate and adaptive arms of the immune system which induce a feed-forward mechanism for inflammation (Murray et al., 2021). Cytolytic NK cells, macrophages, dendritic cells induce tissue damage and vascular dysfunction in PE while activating Th cells through antigen presentation (Deer et al., 2023). Moreover, patients with PE have activated Th cells and activated B cells producing agonistic antibodies against the angiotensin II type 1 receptor (AT1-AA) which lead to antigen specific mechanisms of cell destruction and tissue damage (LaMarca et al., 2016). The communication between immune cells during pregnancy in preeclampsia may also lead to immune memory which could contribute to inflammation postpartum (van Rijn et al., 2016; Kieffer et al., 2017; Brien et al., 2019). This prolonged inflammatory state contributes to hypertension, endothelial dysfunction, and fetal complications (Murray et al., 2021). Because PE presents after the 20th week of gestation, most studies that have investigated inflammation in PE have been limited to studying middle gestation and late gestation periods.

### Dendritic cells and macrophages in preeclampsia

While the initiating factor for uterine inflammation in PE is not fully understood, Mφ and DCs play a role to expand the inflammatory environment in PE. PE placentas have increased invading DCs and macrophages compared to normal placentas (Huang et al., 2008). These uMφ also produce high levels of TNF-α and IFN-γ which contribute to apoptosis of fetal trophoblasts in pregnant mice (Blois et al., 2004). Excessive debris from dying trophoblasts could lead to more internalization and presentation of fetal antigens by Mφ and DCs leading to inflammatory Th subsets in PE. Moreover, PE placentas have shown increased chemokines CCL2, CCL4, CCL7, and CCL20 which promote recruitment of innate immune cells and T cells which could contribute to placental dysfunction in PE. There has been some dispute in the phenotype of uMφs in PE, with some studies showing lower levels of M2s in the placenta while others show higher levels of M2s during PE (Reister et al., 1999; Katabuchi et al., 2003; Schonkeren et al., 2011). These discrepancies have been equated to location of Mφs, citing less localization M2s near trophoblasts and spiral arteries in PE (Faas et al., 2014). Illustrating an important distinction in macrophage location during the pathophysiology of PE. However, there have been few studies to investigate the role of Mφs in the induction of improper spiral artery remodeling in PE; therefore, much more research is needed to better understand how Mφs impact the progression of PE pregnancies.

DCs have been similarly underserved in research of PE pregnancies. In PE there is a higher conventional myeloid DCs to plasmacytoid DC ratio in the circulation (Darmochwal-Kolarz et al., 2003; Wang et al., 2013; Li et al., 2019). These studies also associated the rise in myeloid DCs with increased circulating Th1 and Th17 populations, postulating that myeloid DCs are related to the increased inflammatory Th cell phenotype of PE. However, these studies do not provide as much insight into the placental DC populations in PE. A study by Zhang et al. (2017) showed that there were increased mature dendritic cells in the decidua of PE women, and these DCs had higher expression of a DC specific long non-coding RNA strand that assists in DC maturation by phosphorylating STAT-3. Moreover, a study by Panda et al. (2012) showed that circulating DCs expressed higher levels of TLR3, TLR4, and TLR9 compared to DCs from control patients. These DCs also secreted higher levels of TNF-α, IFN-α, IL-6, and IL-12 measured by flow cytometry. The authors also found that PE DCs were less able to mount inflammatory responses to TLR activation, suggesting that the dysregulation of TLR signaling also impaired DC responses to pathogens. Interestingly, women with PE have increased circulating memory T cells during pregnancy (Chaiworapongsa et al., 2002). Which could suggest that antigen presentation to Th cells leads to development of Th1’s and Th17s during PE but also leads to memory T cell production that persists after a PE pregnancy. However, there is limited research available investigating how DCs become altered in PE and how they are involved in placental dysfunction in PE.

### T helper cells and B cells in preeclampsia

Patients with PE have an increase in Th1/Th2 ratio and an increase Th17/Treg ratio in the placenta and in the circulation (Eghbal-Fard et al., 2019; Romao-Veiga et al., 2022) (Wallace et al., 2014). The location of where Th cells become activated is unknown, but Th cells induce dysfunction at the placental level as well as in other organs and the vasculature (Murray et al., 2021). Th cells are important mediators to spread immune signals throughout the body; and, Th1 cells and Th17 subsets are potent inducers of inflammatory actions and promote cell mediated cytotoxicity (Wang et al., 2020). Interestingly, adoptive transfer of placental Th cells from placentas of PE women induce hypertension in pregnant athymic nude rats (Harmon et al., 2019). Th cell adoptive transfer also lead to increased plasma sflt-1, IL-17, and TNF-α, while also leading to increased expression of pre-pro-endothelin-1 mRNA which is the precursor to the potent vasoconstrictor endothelin-1. This study suggested that placental Th cells contribute to vascular and renal dysfunction during PE. Animal models of PE have been important to help in delineating the effects of T cells on the placenta, the vascular, and the kidney in PE. Adoptive transfer of Th1 cells from the Reduced Uterine Perfusion Pressure (RUPP) model of PE induce hypertension and AT1-AA, placental mitochondrial oxidative stress and FGR, as well as renal oxidative stress during pregnancy (Zenclussen, 2006; LaMarca et al., 2008a). Together these studies illustrate that Th cells activated in response to placental ischemia contribute to dysfunction in the vasculature, placenta, and the kidney during pregnancy. Moreover, adoptive transfer of Th17 cells induce hypertension, placental NK cell activation, circulating NK cell activation, and FGR in pregnant rats (Shields et al., 2018). Further showing that Th17 cells are also able to contribute to placental and vascular dysfunctions in pregnancy with the help of NK cells.

Our lab has shown that Th cells stimulated by placental ischemia from rats or humans induce hypertension, mitochondrial dysfunction, sflt-1, AT1-AA, and other features of PE in pregnant rats (Deer et al., 2021; Reeve et al., 2022). However, blockade of communication between Th cells and B cells prevents the features of PE induced by Th cell adoptive transfer (Cornelius et al., 2015). This indicates that Th cell-B cell communication could be an important feature contributing to the pathophysiology of PE. This is attributed to T cell-B cell communication as an integral component in stimulating B cells to transform into Memory B cells which retain antigen memory long after antigen exposure (Palm and Henry, 2019). There has been much less investigation into the role of B cells in the pathophysiology of PE. B cells can be divided into B1 and B2 subsets. B1 cells are innate-like B cells that are responsible for T cell independent antibody responses while B2 cells are classical B cells that are responsible for T cell dependent antibody responses (Mahajan et al., 2021). In the context of PE, Jensen et al. implicated B1 cells as potential producers of AT1-AA *in vitro* after exposing isolated placental B1 cells to patient serum (Jensen et al., 2012). Our lab recently showed that adoptive transfer of RUPP B2 cells into pregnant rats were able to induce hypertension, AT1-AA production, and NK cell activation during pregnancy (Herrock et al., 2022),therefore, implicating both B1 and B2 subsets in the pathophysiology of PE.

### Soluble immune factors in preeclampsia

Th1 cells are important producers of inflammatory cytokines IFN-γ and TNF-α which are both increased in women with PE (Sheibak et al., 2020). Inhibiting TNF-α in RUPP rats attenuated hypertension, Endothelin-1, oxidative stress, and NK cell activation (LaMarca et al., 2008b; Cunningham et al., 2020). Other studies have infused TNF-α into pregnant animals to see the effects of total TNF-α during pregnancy (Bobek et al., 2015; Ampey et al., 2019; Jayaram et al., 2021). TNF-α alone induces symptoms of PE in animal models including hypertension, FGR, oxidative stress, Endothelin-1 and AT1-AA in pregnancy (LaMarca et al., 2005; Jayaram et al., 2021). But inhibition of TNF-α in multiple models of PE can attenuate hypertension, maternal inflammation, and fetal morbidity (Irani et al., 2010; Travis et al., 2021). PE patients also have increased Th17s and IL-17 (Ding et al., 2019; El Shahaway et al., 2019). IL-17 can cause more Th17 cells which make more IL-17 in a positive-feedback loop which contributes to the inflammation in PE (Travis et al., 2020). Th17s and IL-17 have been connected with oxidative stress, NK cell activation, anti-angiogenic factors, and AT1-AA production in pregnancy, all of which contribute to hypertension and FGR (Cornelius et al., 2013; Travis et al., 2020). Alternatively, IL-17 inhibition prevents hypertension and oxidative stress in placental ischemic rats (Travis et al., 2020) and elicits natural killer cell activation and hypertension further supporting a roll for Th17s and IL-17 in PE (Travis et al., 2019).

Women with PE have activated B cells producing AT1-AA which causes AT1 receptor activation leading to vasoconstriction in afferent arterioles, renal dysfunction and hypertension during pregnancy (Wallukat et al., 1999). AT1-AA acts by binding the AT1 receptor and stimulating it similar to angiotensin II but also work synergistically with endogenous angiotensin II to further increase blood pressure, oxidative stress, production of sflt-1 and Endothelin, indicating that it may exacerbate the activity of endogenous ANGII in PE patients (Brewer et al., 2013). AT1-AA also causes natural killer cell activation, which may be one mechanism responsible for multi-organ dysfunction in PE (Cunningham et al., 2018; Zhai et al., 2022). AT1-AA is also able to induce oxidative stress through NADPH oxidase and activate NF-κB pathway which contribute to vascular stress in PE (Dechend et al., 2003). Moreover, AT1-AA is also increased in the RUPP model of placental ischemia and our lab has shown that directly inhibiting AT1-AA can prevent the PE-like phenotype associated with placental ischemia (Cunningham et al., 2018).

## Immune memory after normal pregnancy

### Adaptive immune memory

After the immune adaptations that happen in pregnancy, the maternal immune system retains memory of paternal antigens in order to facilitate future pregnancies. Following activation, T cells and B cells transform into effector cells and memory cells. While the effector cells are responsible for the short-term immune response, the memory cells retain long-term antigen memory in case of future infections. Paternal antigen memory Treg cells remain after a healthy pregnancy, and persist at least one-year postpartum (Kieffer et al., 2017). Upon initiation of another pregnancy with the same partner, memory Tregs rapidly expand in the circulation upon initiation of a new pregnancy (Tilburgs et al., 2008). These memory Tregs then progressively migrate towards the placenta and promote tolerance of the new fetus (Rowe et al., 2012; Gomez-Lopez et al., 2020). It has been suggested that insufficient memory Treg expansion could contribute to infertility (Jasper et al., 2006) suggesting an important role for Tregs in pregnancy success. Moreover, first-time pregnancies have higher incidence of PE (ACOG, 2022), lower Treg expansion (Rowe et al., 2012), and increased sflt-1 (Bdolah et al., 2014) compared to second-time pregnancies. Furthermore, if a subsequent pregnancy is with a different father than the first, there is no Treg induced protection against PE (Need, 1975). These trends could be explained by the lack of paternal-antigen Tregs, implicating paternal-antigen Tregs to promote fetal tolerance in pregnancy.

### Innate trained immunity

“Trained Immunity” is a relatively new concept in immunology and appears to be unique to the innate immune system. In simple terms, innate immune cells can make epigenetic modifications after encountering an antigen (Netea et al., 2016). This style of memory is in contrast to adaptive immunity, which involves gene recombination events to retain long-term antigen memory. A study by Novakovic et al. (2016) showed that murine Mφs retain epigenetic modifications following *in vitro* exposure to LPS. This study also importantly showed that these epigenetic modifications may be reverse, showing the increased volatility of trained immunity compared to adaptive immunity. Trained immunity is associated with a three-month to one-year lifetime, but there have been cases of trained immunity lasting up to five years (Nankabirwa et al., 2015).

In the context of pregnancy, trained immunity is still in its infancy in terms of investigation.Gamliel et al. (2018) showed that a subset of “pregnancy trained” uNK cells were more readily able to produce IFN-γ and VEGF compared to untrained uNK cells. As stated above, both IFN-γ and VEGF are crucial in implantation and establishing the maternal-fetal interface. Therefore, this study provides crucial insight into direct applications of trained immunity during physiological pregnancy. However, this is one of the only studies to show direct mechanisms of trained immunity in human pregnancy.

## Immune memory and inflammation after a preeclamptic pregnancy

### Adaptive immune memory

A recent study also showed that women with PP-PE have increased inflammatory markers and placental pathologies (Brien et al., 2019). Brien et al. found that patients with PE or PP-PE had increased CD4+ and CD8+ T cells in circulation at time of PE presentation. However, only PP-PE patients had increased circulating Natural Killer T (NKT) cells. NKT cells are an innate-like lymphoid cells that stem from the thymus but share markers of T cells and NK cells (Hashemi et al., 2017). While NKT cells have been implicated in the pathophysiology of PE, this study further implicates NKT cells and inflammation in the pathophysiology of PP-PE. Histological analysis of placentas revealed increased syncytial knots, a morphological marker of dysfunction, suggesting that placental dysfunction is important in PP-PE. This study shows that inflammation may be important in the pathophysiology of PP-PE as well. Therefore, therapies targeting inflammatory mediators may be helpful in treating PE in pregnancy or postpartum.

Following a PE pregnancy, there is a persistent inflammatory profile. Vitoratos et al. (2010) found that PE women had increased plasma TNF-α during pregnancy and this increase in TNF-α persisted three months postpartum. Interestingly, they found that plasma IL-6 was not changed during pregnancy, but plasma IL-6 increased postpartum in PE women compared to NP indicating that inflammation may worsen after a PE pregnancy. It was later found that previously PE women have an exaggerated inflammatory response to the flu vaccine which included increased C reactive protein and IL-18 which are both involved in Th1 responses (van Rijn et al., 2016). This indicates that there are alterations to the normal inflammatory response in women that previously had PE that lasts well into their postpartum period. However, circulating memory Th cells are decreased in patients with a previous PE pregnancy (Kieffer et al., 2019), suggesting that Th dysfunction continues into the postpartum period after PE. Studies of Treg cells in idiopathic pre-term labor patients revealed that decreased functional Tregs is associated with recurrent pregnancy loss (Gomez-Lopez et al., 2020), which could be caused by a sustained inflammatory environment. This is mirrored in subsequent pregnancies following PE, where Tregs do not undergo peripheral expansion nor do they migrate to the uterus (Tsuda et al., 2018). This suggests that dysfunctional memory Tregs may be important in PE recurrence. Interestingly, there is an inverse relationship between memory Tregs and Memory B cells in previously PE patients where lower Tregs is associated with higher Memory B cells (Zeng et al., 2013). Memory B cells from a previously PE pregnancy could continue to secrete AT1-AA and contribute to PE in subsequent pregnancies. Rieber-Mohn et al. found that AT1-AA was increased in serum eight years postpartum of a PE pregnancy (Rieber-Mohn et al., 2018), supporting a role for Memory B cells and AT1-AA not only in PE pathophysiology but also in consequences observed in postpartum PE. Future studies in humans or animal models of PE could investigate the role of memory immune cells to contribute to PE in future pregnancies.

### Innate trained immunity

Unfortunately, there is even less information investigating trained immunity in PE than normal pregnancy. However, an elegant study by Huang et al. showed that both uneventful and PE pregnancies lead to methylation changes in both Th and NK cell populations (Huang et al., 2021). These DNA methylation events are distinct between physiological pregnancies or PE pregnancies, further implicating immunological memory in the increased risk to develop PE following a PE pregnancy. Please refer to the cited article by Huang et al. to get a more thorough understanding of this fascinating immunological adaption to pregnancy.

## Adverse outcomes associated with risk factors for preeclampsia and COVID-19

There are many factors that predispose patients to develop PE in pregnancy. Cardiometabolic disease like obesity, type 2 diabetes (T2D), and hypertension increase the risk for adverse outcomes of pregnancy, including PE (Alston et al., 2022). Each of these diseases have adverse effects on the health of pregnancy, but importantly they are all recognized as states of chronic inflammation (Harrison et al., 2021). Moreover, patients with autoimmune disease like Systemic Lupus Erythematosus, Rheumatoid Arthritis, and Type 1 Diabetes are at an increased risk for developing PE (Tamas et al., 2022). There is also an association between infectious diseases in pregnancy and increased incidence of PE, such as seen in those diagnosed with COVID-19 (Nourollahpour Shiadeh et al., 2017; Lokki et al., 2018).

SARS-CoV-2 infection has been shown to significantly increase complications of pregnancy including PE (Jamieson and Rasmussen, 2022). In 2019, the novel, zoonotic virus SARS-CoV-2 became a global pandemic (Sun et al., 2020). SARS-CoV-2 acts by binding ACE-2 (Angiotensin Converting Enzyme-2) in order to invade host cells (Seyed Hosseini et al., 2020). The virus takes over the host cell, replicates, and eventually spreads to other cells in the body. While its uncertain if pregnant patients have higher risk of contracting COVID (Jamieson and Rasmussen, 2022), pregnant patients with COVID have higher rates of morbidity and mortality (Sayad et al., 2022). Importantly, pregnant patients with COVID have higher rates PE, eclampsia, and preterm birth compared to control (Villar et al., 2021; Wei et al., 2021). Placental samples from a cohort of women with COVID in pregnancy and fetal demise have showed copious placental fibrosis, histiocytic intervillositis, and trophoblast necrosis which has been attributed to COVID induced placental destruction (Jaiswal et al., 2021; Schwartz et al., 2022). Strangely, COVID patients also have increased serum levels of AT1-AA (Rodriguez-Perez et al., 2021). AT1-AA in COVID patients could contribute to post-senescence hypertension in some former COVID patients (Angeli et al., 2022); but, AT1-AA could also contribute to the increased risk for PE in pregnant patients. Moreover, the Renin Angiotensin Aldosterone System (RAAS) must compensate for increased fluid loads during pregnancy (Irani and Xia, 2011; Xia and Kellems, 2011), but dysregulation of RAAS by COVID infection could also contribute to hypertension during pregnancy. Additionally, patients diagnosed with COVID during pregnancy should be monitored postpartum to examine the effects of their uniquely challenging pregnancy.

## Conclusion

In conclusion, developing PE is a major contributor to cardiovascular and immune complications in pregnancy and postpartum. After a PE pregnancy, patients have increased incidence of cardiovascular disease, stroke, and PE in a subsequent pregnancy which could be caused by inflammatory mediators. Moreover, developing severe PE is associated with post-traumatic stress disorders that may discourage patients from having future desired pregnancies (Hoedjes et al., 2011). Recent evidence after the COVID pandemic has shed more light on the relationship between placental ischemia, inflammation, and PE suggesting that immune dysregulation could be a major contributor to PE development. Yet, more studies are needed to investigate the mechanisms involved to cause cardiovascular disease or PE recurrence in previously PE patients. Immune memory may contribute to cardiovascular disease or PE recurrence following a PE pregnancy, but more studies could investigate mechanisms of inflammation to induce these features in animal models of PE. Modulators of inflammation have shown promise to lessen maternal disease in models of PE, but longitudinal studies could show efficacy of these agents in the postpartum period. Importantly, investigators must continue to reveal important pathophysiological components that lead to the onset of PE so that we may better serve patients stricken by this terrible disease.
